# Effects of a behavioral and health literacy intervention to reduce sugar-sweetened beverages: a randomized-controlled trial

**DOI:** 10.1186/s12966-016-0362-1

**Published:** 2016-03-22

**Authors:** Jamie M. Zoellner, Valisa E. Hedrick, Wen You, Yvonnes Chen, Brenda M. Davy, Kathleen J. Porter, Angela Bailey, Hannah Lane, Ramine Alexander, Paul A. Estabrooks

**Affiliations:** Department of Human Nutrition, Foods, and Exercise, Virginia Tech, 1981 Kraft Drive (0913), ILSB 23, Blacksburg, VA 24061 USA; Department of Agricultural and Applied Economics, Virginia Tech, Blacksburg, VA 24061 USA; School of Journalism, University of Kansas, Lawrence, KS 66045 USA; Department of Health and Exercise Science, Rowan University, Glassboro, NJ 08028 USA; Department of Health Promotion, Social & Behavioral Health, University of Nebraska Medical Center, Omaha, NE 68198-4365 USA

**Keywords:** Beverages, Health literacy, Behavioral research, Randomized controlled trial, Rural population

## Abstract

**Background:**

Despite excessive consumption of sugar-sweetened beverages (SSB), little is known about behavioral interventions to reduce SSB intake among adults, particularly in medically-underserved rural communities. This type 1 effectiveness-implementation hybrid RCT, conducted in 2012–2014, applied the RE-AIM framework and was designed to assess the effectiveness of a behavioral intervention targeting SSB consumption (SIP*smart*ER) when compared to an intervention targeting physical activity (MoveMore) and to determine if health literacy influenced retention, engagement or outcomes.

**Methods:**

Guided by the Theory of Planned Behavior and health literacy strategies, the 6 month multi-component intervention for both conditions included three small-group classes, one live teach-back call, and 11 interactive voice response calls. Validated measures were used to assess SSB consumption (primary outcome) and all secondary outcomes including physical activity behaviors, theory-based constructs, quality of life, media literacy, anthropometric, and biological outcomes.

**Results:**

Targeting a medically-underserved rural region in southwest Virginia, 1056 adult participants were screened, 620 (59 %) eligible, 301 (49 %) enrolled and randomized, and 296 included in these 2015 analyses. Participants were 93 % Caucasian, 81 % female, 31 % ≤ high-school educated, 43 % < $14,999 household income, and 33 % low health literate. Retention rates (74 %) and program engagement was not statistically different between conditions. Compared to MoveMore, SIP*smart*ER participants significantly decreased SSB kcals and BMI at 6 months. SIP*smart*ER participants significantly decreased SSB intake by 227 (95 % CI = −326,−127, *p* < 0.001) kcals/day from baseline to 6 months when compared to the decrease of 53 (95 % CI = −88,−17, *p* < 0.01) kcals/day among MoveMore participants (*p* < 0.001). SIP*smart*ER participants decreased BMI by 0.21 (95 % CI = −0.35,−0.06; *p* < 0.01) kg/m^2^ from baseline to 6 months when compared to the non-significant 0.10 (95 % CI = −0.23, 0.43; NS) kg/m^2^ gain among MoveMore participants (*p* < 0.05). Significant 0–6 month effects were observed for about half of the theory-based constructs, but for no biological outcomes. Health literacy status did not influence retention rates, engagement or outcomes.

**Conclusions:**

SIP*smart*ER is an effective intervention to decrease SSB consumption among adults and is promising for translation into practice settings. SIP*smart*ER also yielded small, yet significant, improvements in BMI. By using health literacy-focused strategies, the intervention was robust in achieving reductions for participants of varying health literacy status.

**Trial registration:**

Clinicaltrials.gov; ID: NCT02193009.

## Background

Health concerns surrounding the excessive consumption of sugar-sweetened beverages (SSB) is arguably one of the most publicized current public health issues. A widely accepted description of SSB includes all carbonated beverages with added sugar (e.g., soda), fruit drinks with added sugar, sports drinks, and other drinks with added sugar such as sweetened tea and coffee. SSB are the largest contributor to added sugar intake and contribute approximately 7 % of total energy intake for United States adults [1]. Of additional importance are the significantly higher SSB intake patterns among rural adults and among adults with lower health literacy (HL) skills [2, 3].

There is strong scientific evidence indicating associations between SSB and numerous adverse health issues such as obesity, diabetes, cardiovascular disease, dental caries, and oral health [4–8]. Although notable emphasis has been placed on behavioral intervention strategies targeting SSB reduction among children and adolescents, there has been little attention on developing evidence-based behavioral interventions targeting SSB consumption among adults [9], particularly among low socioeconomic, rural adults who are at greater risk for high SSB consumption. Likewise, medically underserved populations have less access to health care services and fewer opportunities to participate in and benefit from health behavior research. Engaging underserved populations in behavioral interventions is central to reducing disparities and the prevalence of adverse health outcomes [10].

Similar to the link between SSB and adverse health conditions, poor HL skills have consistently been linked with poorer health outcomes [11, 12]. Health literacy can be defined as “an individual’s capacity to obtain, process, and understand basic health information and services needed to make appropriate health decisions [13].” Despite the emergence of experimental approaches to address HL skills, the influence of participant HL status on program effectiveness is not well understood and few studies have explored moderation effects by HL status [12, 14].

The Talking Health trial was developed to address these gaps in the literature and to target needs of the medically-underserved Appalachian region of rural southwest Virginia [15]. Unfortunately, this targeted region consumes over three times the national average intake of SSB [16]. There are also notable socioeconomic (median income, percent population below poverty, educational achievement, etc.) and literacy proficiency disparities within this region, as compared to state and national averages [17, 18].

The Talking Health trial was designed to determine the effectiveness of a 6 month intervention aimed at decreasing SSB consumption (SIP*smart*ER) when compared to a matched-contact intervention group targeting physical activity (PA) behaviors (MoveMore). The Theory of Planned Behavior (TPB) and HL strategies were applied in all phases of intervention planning, implementation, and evaluation [19]. The study was designed as a type 1 effectiveness-implementation hybrid randomized-controlled trial that was guided by the RE-AIM (reach, effectiveness, adoption, implementation, maintenance) planning and evaluation framework [15, 20]. A type 1 hybrid trial has primary objectives related to testing intervention effectiveness and secondary objectives aimed at better understanding the context for implementation [15]. The purpose of this paper is to report on changes in SSB consumption, PA behaviors, theoretical antecedents of behavior change, quality of life, media literacy, anthropometric, and biological outcomes. A secondary purpose is to determine the robustness of effects by participant baseline HL status when considering retention rates, program engagement, and outcomes. Finally, descriptive data is provided across RE-AIM dimensions of reach and implementation.

## Methods

This community-based randomized-controlled trial occurred in an eight-county rural region in southwest Virginia (i.e., Lee, Giles, Pulaski, Washington, Grayson, Wise, Wythe, and Montgomery). All included counties are federally designated as medically underserved [21], and considered rural by population density metrics [22]. A variety of active (e.g., recruitment at health departments) and passive (e.g., flyers, newspaper ads, word of mouth) recruitment strategies were used throughout the targeted counties between March 2012 and May 2014. Eight counties/cohorts were spaced approximately 3 months apart and averaged 39 participants/cohort (range 31–53). The first cohort intervention period was from May-October 2012 and the final from May-November 2014.

Eligibility criteria included English-speaking adults ≥18 years of age, with self-reported consumption of ≥200 SSB kcals/day [23], no self-reported contraindications for physical activity [24], regular access to a telephone, and not concurrently enrolled in another nutrition or physical activity program. Also, in efforts to minimize cross-contamination between intervention groups, only one member per household was allowed to enroll in the trial. Upon completing the baseline assessment, enrolled participants were randomly assigned to SIP*smart*ER (*n* = 155) or MoveMore (*n* = 146). Randomization occurred at the individual level. An equal number of sealed envelopes were prepared indicating SIP*smart*ER or MoveMore conditions and each participant drew an envelope to indicate their randomized condition. Locations for data collection and intervention activities varied across cohorts but included centrally located venues within each community (e.g., public health buildings, Extension offices, churches).

### Ethics approval

All study procedures were approved by the Virginia Tech Institutional Review Board, and participants provided written informed consent to participate in the study. To compensate their time to complete data assessments, $25 and $50 gift cards were provided at baseline and 6 month assessments, respectively.

### Interventions

SIP*smart*ER targeted decreasing SSB consumption, with the primary goal of achieving the SSB recommendation of less than 8 fluid ounces per day [6, 25]. To sufficiently target SSB reduction, participants were educated on recommendations for all beverage categories (e.g., water, noncalorically sweetened beverages, milk) [25]. A pragmatic approach was taken when developing a comparison condition that was matched for contact and structure, but focused on a behavior independent of SSB consumption. This approach ensured that all study participants in these medically-underserved counties had an opportunity to benefit from study participation [26]. As such, the comparison condition, MoveMore targeted PA promotion, with the primary goal of achieving 150 min of moderate-intensity aerobic activity and doing muscle-strengthening activities on two or more days per week [27].

Several formative research phases guided development of the culturally-sensitive SIP*smart*ER intervention [16, 28, 29]; and MoveMore was adapted from a previous research tested group-based PA intervention [30–32]. Prior to launching the Talking Health trial, a 5 week randomized-controlled pilot test was used to evaluate participant feedback on intervention content and structure, as well as understand the potential reach and preliminary effect sizes [29]. The final 6 month intervention structure, informed by the preliminary work, included three small-group classes, one live teach-back call, and 11 Interactive Voice Response (IVR) calls. SIP*smart*ER and MoveMore conditions were matched in duration and contact. Each of the small group classes were 90–120 min in duration, and delivered during weeks one, six, and seventeen. Participants who missed a class were mailed a packet that outlined key content information and then a research assistant called participants to verbally review and reinforce the content, using a semi-structured script. Approximately 1 week following the first class (or missed class call), a scripted teach-back call occurred, lasting an average of 18.6 (*SD* = 5.6) minutes. Participants were asked to teach-back key concepts from the first class and to explain how they tracked their behaviors and calculated weekly averages. When recalled incorrectly, participants were given correct answers and offered additional opportunities to recall concepts correctly [33]. Participants also received 11 IVR calls, weekly for the first 3 weeks and then bi-weekly for the remainder of the intervention. Each IVR call, lasting an average of 6.9 (*SD* = 1.9) minutes, reinforced key intervention messages, provided new content, and led participants through a personal action planning procedure [34–36].

A comprehensive overview of the intervention structure, theoretical constructs, and key learning objectives for SIP*smart*ER and MoveMore are published elsewhere [37]. In brief, the foundational program elements including the TPB [19, 38–41] and concepts related to HL, media literacy, and numeracy [42–45]. Clear communication techniques are embedded throughout the interventions, including activity approaches (e.g., hands-on demonstrations, pictorial information); materials with simplified language; teach-back strategies to promote comprehension of learning objectives [33]; and non-written reinforcement of key intervention messages (i.e., IVR calls). Intervention content is aimed at building HL skills related to numeracy [41] and to interpreting SSB- and PA-specific media messages [46] as well as self-monitoring skills (e.g., personal action planning and behavior tracking) [47].

Three masters-level research staff (i.e., MPH, MS/RD, MS/MCHES) and two PhD investigators with expertise in media literacy delivered the classes. Trained graduate research assistants provided additional class support and completed the teach-back calls.

Implementation data was tracked via detailed bi-monthly research meeting minutes and the IVR system generated reports. Program engagement was tracked systematically in SPSS statistical analyses software and operationalized as attending small group classes or completing missed call, completing the teach-back call, and completing the IVR calls.

### Measures

Assessment included interviewer-administered questionnaires, computer-audio assisted questionnaires, and anthropometric and biological measures. Interviewer-administered validated questionnaires included: 1) the Newest Vital Sign, a 6-item questionnaire, to assess HL based on the nutrition facts panel [48], 2) three non-consecutive 24 h dietary recalls over a 2 week period, including 2 weekdays and 1 weekend day [49], and 3) Godin Leisure Time Exercise Questionnaire to estimate time spent in moderate, vigorous, and strength training physical activity over the past 7 days [50]. Computer-audio assisted questionnaires included: 1) BEVQ-15, a validated food-frequency instrument that assesses past month beverage consumption [51, 52], 2) Stanford Leisure-Time Activity Categorical Item, a 1-item PA questionnaire which asks participants to select one of six statements that describes their PA level over the past month [53], 3) TPB questions related to SSB consumption (20-items) and physical activity (20-items), which addressed attitudes, subjective norms, perceived behavioral control, and behavioral intentions [28, 54], 4) media literacy scale to address perceptions of media and advertisements related to SSB (19-items) [55], and 5) quality of life to assess physical, mental, and overall health (4-items) [56]. Anthropometric and biological assessments included: 1) weight measured without shoes and light clothing using a calibrated digital Tanita scale (Model: 310GS), 2) height measured with a research-grade stadiometer (at baseline only), 3) blood pressure following American Heart Association guidelines and measured with an automated oscillometric device (OMRON, Model:HEM-907XL) [57], and 4) fasting blood samples obtained via routine finger sticks using a One Touch Fine Point Lancet (Lifescan, Johnson & Johnson Company). A CardioChek system was used to determine blood glucose, cholesterol, and triglyceride concentrations [58].

### Statistical analysis

All data were entered into SPSS statistical analyses software (version 21.0, 2012, International Business Machines Corporation, Pittsburgh, PA) and validated scoring procedures were applied to compute outcome variable scores. The Nutrition Data System for Research software (*NDS-R,* University of Minnesota, 2011) was used to analyze the 24 h dietary recall data and to calculate average energy intake (i.e., total kilocalorie intake reported at each recall divided by the number of days of provided recalls). Participant engagement was calculated as the proportion of program events completed and included proportion of small group classes or missed class calls attended/completed out of three, proportion of teach-back call completed out of one, and proportion of IVR calls completed out of 11. Descriptive statistics were used to summarize baseline demographic characteristics and engagement rates. Chi-square tests of association or Fisher’s exact tests (categorical variables) and ANOVA tests (continuous variables) were used to compare demographics and engagement rates between conditions.

Multilevel mixed-effects linear regressions and moderation analysis were performed using Stata software to account for clustering of individuals within county cohorts (version 13, 2013, StataCorp LP, College Station, TX). Our original analysis included 6 month present at follow-up (completers only), as well as intention-to-treat using baseline value carried forward (which is also the last-observation-carried-forward since there are just two observations of the participants during the program duration) and intention-to-treat using multiple imputations to account for missing data [59, 60]. All three analytic procedures yielded similar results for the primary outcomes, with the expected largest effect sizes found in the present at follow-up analysis. Therefore, the most conservative approach, intention-to-treat using baseline value carried forward, is reported in this manuscript.

The mixed-effect models controlled for individual baseline characteristics, dummies of time and condition, and a time by condition dummy interaction. All models calculated robust standard errors for county/cohort cluster. The baseline covariates controlled in the models were chosen a priori and included age, gender, race/ethnicity, income, education level, HL level, employment status, number of children, smoking status, and BMI [61]. The potential for HL to moderate intervention effects was tested using mixed-effect models with two- and three-way interactions among time, condition, and HL status added. The HL was based on standardized scoring for the NVS (0–3 correct answers = low HL; 4–6 correct answers = high HL). The coefficient of the three-way interaction is the moderation effect of HL status on the relative treatment effects between SIP*smart*ER and MoveMore conditions.

As shown in Table 1, there are no statistical significant differences between conditions which confirms the validity of randomization. Therefore, we also report Cohen’s d effect sizes for the relative 6 month mean treatment effects between the two conditions for the main outcomes [62]. The local effect size Cohen’s f^2^ yielded similar conclusions as well. Our study was powered to detect a small effect size of 0.34 for 0–6 month changes in SSB intake between the SIP*smart*ER and MoveMore conditions (i.e., 80 % power, 0.05 type 1 error).

**Table 1:** Baseline sample characteristics, by randomized condition assignment, n (%) unless otherwise noted

Characteristic	Total Sample	SIP*smart*ER	MoveMore	Test statistic *p*-value
	(*n* = 296)	(*n* = 151)	(*n* = 145)	
Age				
Age (years), M (SD)	42.1 (13.4)	41.7 (13.4)	42.4 (13.3)	F = 0.17 *p* = 0.68
Age 18–24	37 (13)	22 (15)	15 (10)	χ^2^ = 1.35 *p* = 0.72
Age 24–44	134 (45)	67 (44)	67 (46)	
Age 45–64	118 (40)	59 (39)	59 (41)	
Age ≥65	7 (2)	3 (2)	4 (3)	
Gender				
Male	56 (19)	30 (20)	26 (18)	χ^2^ = 0.18 *p* = 0.67
Female	240 (81)	121 (80)	119 (82)	
Race				
Caucasian	275 (93)	137 (91)	138 (95)	χ^2^ = 2.22 *p* = 0.14
African American	13 (4)	10 (6)	3 (2)	
More than one race	7 (2.5)	3 (2)	4 (3)	
Other	1 (0.5)	1 (1)	0 (0)	
Ethnicity				
Hispanic/Latina	3 (1)	2 (1)	1 (0.5)	χ^2^ = 0.48 *p* = 0.79
Income				
≤ 14,999	126 (43)	69 (46)	57 (39)	χ^2^ = 6.89 *p* = 0.08
15,000–34,999	94 (32)	52 (35)	42 (29)	
35,000–54,999	39 (13)	18 (12)	21 (15)	
≥ 55,000	37 (12)	12 (8)	25 (17)	
Employment Status				
Employed full or part time	144 (49)	67 (44)	77 (53)	χ^2^ = 2.26 *p* = 0.13
Unable to work/on disability	51 (17)	31 (21)	20 (14)	χ^2^ = 2.35 *p* = 0.13
Number of Children				
At least 1 child in household	149 (50)	77 (49)	72 (50)	χ^2^ = 0.05 *p* = 0.82
No children in household	147 (50)	74 (51)	73 (50)	
Education Level				
</=High school graduate	93 (31)	49 (32.5)	44 (30)	χ^2^ = 0.15 *p* = 0.70
Some college or greater	203 (69)	102 (67.5)	101 (70)	
Anthropometry				
Weight (kg), M (SD)	90.6 (25.4)	90.7 (26.4)	90.4 (24.3)	*F* = 0.01 *p* = 0.93
BMI (kg/m^2^), M (SD)	33.0 (9.1)	33.3 (9.3)	32.7 (9.0)	*F* = 0.24 *p* = 0.62
Underweight (≤18.4)	5 (2)	3 (2)	2 (1.5)	χ^2^ = 1.45 *p* = 0.92
Normal weight (18.5–24.9)	59 (20)	28 (19)	31 (21)	
Overweight (25–29.9)	64 (22)	34 (23)	30 (21)	
Obese (≥30)	168 (57)	86 (57)	82 (57)	
Obese class 1 (30–34.9)	60 (20)	30 (20)	30 (21)	
Obese class 2 (35–39.9)	43 (15)	20 (13)	23 (16)	
Obese class 3 (≥40)	65 (22)	36 (24)	29 (20)	
Current Smoker	94 (32)	53 (35)	41 (28)	χ^2^ = 1.59 *p* = 0.21
Health Literacy (HL) Status ^a^				
High likelihood or Possibility of limited HL (score 0–3)	97 (33)	55 (36)	42 (29)	χ^2^ = 1.87 *p* = 0.18
Adequate HL (score 4–6)	199 (67)	96 (64)	103 (71)	

F test were used to compare means across conditions and χ^2^ testes were used to compare proportions across the conditions. Cells do not always equal 100 % due to rounding

*M* Mean, *SD* Standard Deviation

^a^Health literacy was assessed using the validated Newest Vital Sign

## Results

### Study reach

**Fig. 1:**
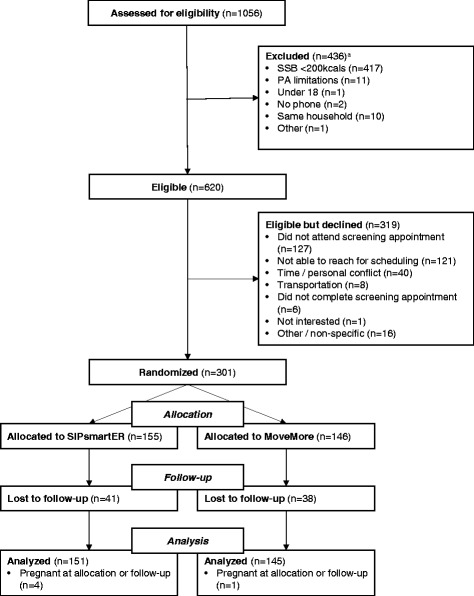
Study flow chart for the Talking Health trial. ^a^Some potential participants may have been excluded for >1 eligibility criteria

In total, 1056 participants were screened, 620 were eligible (59 %), and 301 (49 %) enrolled (Fig. 1). Retention was 74 % at 6 month follow-up and was not statistically different between conditions. Five women reported being pregnant at baseline and/or the 6 month follow-up and were excluded from analysis. The 296 adult participants (mean age 42.1 ± 13.4 years) included in the analysis were 93 % Caucasian, 81 % female, 31 % ≤ high-school educated, 43 % < $14,999 household income, and 33 % with low HL (Table 1). The mean body mass index (BMI) was 33.0 ± 9.1 kg/m^2^ and the majority of participants were overweight (21.5 %) or obese (57.0 %). There were no significant demographic differences between conditions.

When compared to average US Census data across these counties [17], our enrolled participants had a substantially lower mean income (enrolled = $23,173, Census = $48,105), yet had somewhat higher educational attainment (enrolled = 68 % ≥ HS, Census = 58 % ≥ HS) and had a considerably higher proportion of females (enrolled = 81 %, Census = 48 %). There were no differences in terms of race (enrolled = 93 % Caucasian, Census = 94 % Caucasian).

### Implementation and program engagement

When considering both SIP*smart*ER and MoveMore, a total of 96 classes (2 sessions of each class per cohort per condition) were planned and delivered. The overall receipt rate of intervention content was 70 %, with 58 % average class attendance and 12 % missed class call completion. Teach-back call attempts were made to all 296 participants, and 70 % of the participants completed the call. Finally, 11 IVR calls were attempted for all participants and the average completion rate was 51 %. There were no statistically significant differences in engagement rates between the two conditions.

### Effectiveness

For the primary outcomes, daily kilocalories and fluid ounces of SSB consumption, 6 month effects were statistically significant between conditions and moderate in magnitude (Table 2). For example, SIP*smart*ER participants significantly decreased SSB intake by 227 (95 % CI = −326,−127, *p* < 0.001) kcals/day from baseline to 6 months when compared to the decrease of 53 (95 % CI = −88,−17, *p* < 0.01) kcals/day among MoveMore participants (*p* < 0.001) (Cohen’s d effect size = 0.55). The minutes/day of moderate to vigorous PA revealed a small and statistically significant time effect for the MoveMore condition, but not a statistically significant between condition effect (Cohen’s D effect size = 0.23). However, the number of minutes of strength training activity revealed a moderate and significant effect between conditions (Cohen’s D effect size = 0.43).

**Table 2:** Changes in self-reported outcomes by treatment condition (*n* = 296)

Variable	SIP*smart*ER			Move More			Relative effects between conditions^b^
	Base-line^a^	6 month^a^	Adjusted change baseline to 6 month^b^	Base-line^a^	6 month^a^	Adjusted change baseline to 6-month^b^	
SSB, kcals/day	496 (374)	268 (297)	−227 (−326, −127)***	377 (287)	325 (319)	−53 (−88, −17)**	−174 (−276, −71)***
SSB, fluid ounces/day	43 (31)	24 (24)	−19 (−28, −10)***	33 (24)	28 (27)	−5 (−7,−2)***	−14 (−23, −6)**
Total energy intake, kcals/day	1973 (1095)	1696 (1098)	−277 (−425, −128)***	1780 (647)	1737 (688)	−43 (−134, 48)	−234 (−444, −24)**
MVPA, min/day	40 (50)	43 (51)	3 (−12, 18)	39 (51)	54 (51)	15 (6, 24)**	−12 (−28, 5)
Strength Training PA, min/day	13 (60)	11 (36)	−3 (−9, 3)	7 (28)	24 (56)	17 (7, 28)**	−20 (−32, −7)**
Stanford Leisure-Time Activity Categorical Item (L-CAT)^c^	0.57 (0.88)	0.70 (1.01)	0.15 (−0.08, 0.37)	0.67 (1.07)	1.06 (1.06)	0.40 (0.28,0.51)***	−0.25 (−0.46, −0.04)*
Quality of Life, # unhealthy days/month	8.4 (9.3)	7.3 (8.0)	−1.2 (−2.4, −0.1)*	7.3 (7.5)	7.1 (7.2)	−0.1 (−0.8, 0.5)	−1.1 (−2.23, 0.02)
SSB Media Literacy^d^	110.2 (17.3)	118.4 (15.9)	8.2 (6.5,9.9) ***	113.6 (11.7)	116.5 (11.1)	2.9 (2.0, 3.8) ***	5.3 (2.9, 7.6)***
TPB-SSB Attitudes^e^	4.5 (1.0)	5.2 (1.1)	0.7 (0.6, 0.9)***	4.6 (1.0)	4.8 (1.1)	0.2 (0.1, 0.3)***	0.5 (0.3, 0.7)***
TPB-SSB Subjective Norms^e^	4.8 (1.3)	5.1 (1.4)	0.3 (0.1, 0.5)**	4.7 (1.3)	4.9 (1.3)	0.2 (0.0, 0.5)*	0.0 (−0.3, 0.4)
TPB-SSB Perceived Behavioral Control^e^	5.2 (1.5)	5.7 (1.3)	0.6 (0.3, 0.8)***	5.5 (1.2)	5.8 (1.1)	0.3 (0.0, 0.5)*	0.3 (0.0, 0.6)*
TPB-SSB Behavioral Intentions^e^	4.7 (1.7)	5.6 (1.6)	1.0 (0.6, 1.3)***	4.8 (1.5)	5.0 (1.5)	0.3 (0.1, 0.4)*	0.7 (0.3, 0.9)***
TPB-PA Attitudes^e^	5.5 (1.0)	5.5 (1.1)	−0.0 (−0.1, 0.1)	5.5 (0.9)	5.6 (0.8)	0.1 (−0.0, 0.2)	−0.1 (−0.3, 0.1)
TPB-PA Subjective Norms^e^	5.5 (1.3)	5.4 (1.3)	−0.1 (−0.3, 0.1)	5.4 (1.10)	5.5 (1.1)	0.1 (−0.1, 0.2)	−0.2 (−0.4, 0.1)
TPB-PA Perceived Behavioral Control^e^	5.5 (1.2)	5.5 (1.3)	0.1 (−0.1, 0.2)	5.4 (1.2)	5.6 (1.1)	0.2 (0.1, 0.4)**	−0.1 (−0.3, 0.1)
TPB-PA Behavioral Intentions^e^	4.9 (1.3)	4.7 (1.5)	−0.1 (−0.4, 0.1)	4.8 (1.2)	5.1 (1.1)	0.4 (0.2, 0.5)***	−0.5 (−0.8, −0.2)**

Within condition and between condition statistical significance indicated by bold face asterisks: *p < 0.05, **p < 0.01, ***p < 0.001

*SS* SIP*smart*ER condition, *MM* MoveMore condition, *SSB* Sugar-sweetened beverages, *PA* Physical Activity, *TPB* Theory of Planned Behavior

^a^ Means (Standard Deviations) are not adjusted for covariates

^b^ Models controlled for baseline covariates including age, gender, race/ethnicity, income, education level, health literacy level, employment status, number of children, smoking status, and BMI. The 95 % confidence intervals are also adjusted to be cohort robust. Analytic procedures uses intention-to-treat last observation carried forward imputations

^c^ L-CAT scale range: 0 = low, 5 = high

^d^ Media literacy scale range: 19 = low, 133 = high

^e^ Theory of Planned Behavior scale range: 1 = low, 7 = high

Significant between condition effects were also found for total energy intake, Stanford Leisure-Time Activity Categorical Item, SSB media literacy, TPB-SSB attitudes, TPB-SSB perceived behavioral control, TPB-SSB behavioral intentions, and TPB-PA behavioral intentions and each in the directions hypothesized (Table 2). The SIP*smart*ER condition significantly improved quality of life over 6 months; however, between condition effects were not significant. Our results did not show evidence of statistically significant between condition effects for TPB-SSB subjective norms, TPB-PA attitudes, TPB-PA subjective norms, or TPB-PA perceived behavioral control. Conversely, significant time effects were found in the MoveMore condition for TPB-PA attitudes (present at follow-up analysis only) and TPB-PA perceived behavioral control.

A between condition effect was also significant for BMI and weight (Table 3). For example, SIP*smart*ER participants decreased BMI by 0.21 (95 % CI = −0.35,−0.06; *p* < 0.01) kg/m^2^ from baseline to 6 months when compared to the non-significant 0.10 (95 % CI = −0.23, 0.43; NS) kg/m^2^ gain among MoveMore participants (*p* < 0.05). There were no between condition effects for other biological and blood pressure outcomes.

**Table 3:** Changes in anthropometric, biological, and blood pressure outcomes by treatment condition (*n* = 296)

Variable	SIP*smart*ER			Move More			Relative effects between conditions^b^
	Base-line^a^	6 month^a^	Adjusted change baseline to 6 month^b^	Base-line^a^	6 month^a^	Adjusted change baseline to 6 month^b^	
Body Mass Index (BMI), kg/m^2^	33.26 (9.28)	33.05 (9.23)	−0.21 (−0.35, −0.06)**	32.74 (8.98)	32.84 (9.08)	0.10 (−0.09, 0.30)	−0.31 (−0.55, −0.07)*
Weight, kg	90.7 (26.4)	90.2 (26.4)	−0.5 (−0.9, −0.0)*	90.4 (24.3)	90.5 (24.7)	1.0 (−0.2, 0.4)	−0.6 (−1.1, −0.1)*
Cholesterol, mg/dL	162.9 (38.2)	155.5 (36.6)	−7.4 (−14.3, − 0.5)*	168.7 (36.4)	163.3 (32.4)	−5.4 (−13.2, 2.4)	−2.0 (−9.4, 5.3)
Low-density lipoprotein (LDL) cholesterol, mg/dL	95.3 (32.2)	90.4 (29.5)	−4.9 (−9.2, −0.7)*	99.9 (31.7)	95.2 (30.7)	−4.8 (−9.2, −0.4)*	−0.1 (−5.6, 5.4)
High-density lipoprotein (HDL) cholesterol, mg/dL	45.5 (15.2)	43.0 (15.0)	−2.6 (−5.3, 0.2)	46.5 (14.9)	45.0 (16.0)	−1.6 (−4.8, 1.7)	−1.0 (−4.4, 2.4)
Triglycerides, mg/dL	126.2 (77.3)	125.0 (75.4)	−1.2 (−10.6, 8.2)	128.6 (75.6)	127.5 (70.2)	−1.1 (−8.5, 6.4)	−0.1 (−12.4, 12.2)
Glucose, mg/dL	80.3 (26.2)	77.1 (26.7)	−3.2 (−6.6, 0.2)	77.1 (24.0)	74.8 (18.0)	−2.4 (−8.0, 3.2)	−0.8 (−3.6, 2.0)
Systolic blood pressure, mm Hg	123.3 (18.0)	122.7 (17.6)	−0.6 (−3.9, 2.7)	120.7 (13.3)	121.0 (15.4)	0.3 (−2.6, 3.2)	−0.9 (−3.6, 1.8)
Diastolic blood pressure, mm Hg	79.5 (11.7)	80.0 (12.3)	0.54 (−1.0, 2.1)	79.7 (10.6)	79.8 (11.0)	0.1 (−1.3, 1.5)	0.4 (−0.8, 1.6)

Within condition and between condition statistical significance indicated by bold face asterisks: *p < 0.05, **p < 0.01, ***p < 0.001

SS = SIP*smart*ER condition; MM = MoveMore condition

^a^ Means (Standard Deviations) are not adjusted for covariates

^b^ Models controlled for baseline covariates including age, gender, race/ethnicity, income, education level, health literacy level, employment status, number of children, smoking status, and BMI. The 95 % confidence intervals are also adjusted to be cohort robust. Relative effects between conditions using intention-to-treat last observation carried forward imputations procedure

### Robustness of effects: health literacy (HL) status

Baseline HL status did not significantly influence 6 month retention rates (low HL = 79 ± 41 %, high HL = 72 ± 45 %; *p* = 0.56) or class engagement rates (low HL = 2.14 ± 1.03, high HL = 1.97 ± 1.15; *p* = 0.20), teach-back call completion (low HL = 0.76 ± 0.43, high LH = 0.66 ± 0.48; *p* = 0.07), or IVR completion (low HL = 5.93 ± 4.62, high HL = 5.45 ± 4.31; *p* = 0.36). Furthermore, baseline HL status did not moderate any of the primary or secondary outcomes. For example, among the SIP*smart*ER condition, the 6 month SSB kcal reduction between participants with low HL and participants with high HL was not statistically significant (*p* = 0.21) and similar for MoveMore condition (*p* = 0.67). Between the low HL and high HL groups, the relative treatment effect between two conditions was also not statistically significant (*p* = 0.31).

## Discussion

The Talking Health trial used was the first to integrate behavioral theory and HL concepts to reduce SSB intake in a rural low socioeconomic area that has some of the highest SSB consumption patterns in the country. The intervention, SIP*smart*ER, demonstrated a moderate to large effect size for reducing SSB intake, when compared to MoveMore. The magnitude of effect was similar to the Choose Healthy Options Consciously Everyday (CHOICE) trial that randomly assigned participants to replace caloric beverages with either water or diet beverages (~200-250 kcals/day reduction) [9]. Of note, the 6 month CHOICE intervention included providing replacement beverages and monthly support meetings to address behavioral strategies and promote adherence to the water or diet replacement beverages.

The comparison to the CHOICE intervention provides a number of practical conclusions and areas of further inquiry [9]. First, unlike SIP*smart*ER, the CHOICE included the provision of replacement beverages—a practice that may not be generalizable to real-world settings who serve rural, low SES regions. Second, SIP*smart*ER was delivered using a mixture of traditional health education (i.e., small group sessions) along with HL (e.g., teach-back and clear communication) and automated scalable (i.e., IVR) strategies, whereas CHOICE included monthly group behavioral classes, monthly weigh-ins, and access to a study website for weekly beverage and weight monitoring. Third, Talking Health participants (31 % ≤ high school education) were considerably less educated than those in the CHOICE trial (7.5 % ≤ high school education). However, the average weight loss among CHOICE trial participants was larger than our trial. This is attributable in part, to the eligibility criteria for overweight or obese BMI in the CHOICE trial. In our trial, weight loss was not a primary focus, nor was weight status an eligibility criterion (e.g., 22 % of enrolled participants had a BMI classification of under or of normal weight). Although reductions in BMI and weight were statistically significant among SIP*smart*ER participants, the clinical significance is relatively small. Nonetheless, this is important data since weight-related outcomes from randomized controlled trials and experimental studies targeting SSB behaviors is limited, especially among adults [7, 63–68]. Given that changes in SSB occurred gradually over the 6 month intervention, data from the on-going 12 month maintenance phase of this trial are needed to make firm conclusions about meaningful reductions in weight and BMI [37].

Secondary to our assessment of SIP*smart*ER, we also documented significant improvements in PA for participants assigned to MoveMore. When compared to the intervention that provided the content basis for MoveMore, the magnitude of effect (small to moderate) in our study was smaller than that found in the original MoveMore intervention (moderate to large effects). However, there are a number of differences between the two trials in terms of target population (members of an HMO vs rural, low SES), primary outcome (moderate PA vs moderate to vigorous and strength training), and duration of the intervention (3 vs 6 months) [30–32]. Further, when compared to a recent meta-analysis of 358 reports and the average mean effect size on interventions designed to increase physical activity among healthy adults, the increase was greater for participants in MoveMore [69]. In line with recommendations for more pragmatic research, our trial highlights an innovative design where participants randomized to the primary outcome group and the comparison group were allowed the opportunity to benefit [26, 70]. Our decision to include a matched contact treament condition was driven by the ethical irresponsibility of enrolling yet providing no treatment to the targeted medically underserved participants, the amount of resources to access and recruit rural participants only to offer a placebo control protocol group, and concerns with attrition of a placebo control.

The improvement in quality of life among SIP*smart*ER participants, as well as the lack of a between group difference, provides a participant centered check and implies that the interventions were not causing negative side effects. Related to the TPB variables, prior literature has consistently established that behavioral intentions are the strongest predictor of behavior [38–41]. In our trial, both the SIP*smart*ER and MoveMore conditions resulted in significant between group improvements in behavior-specific intentions. In general, improvements in attitudes, subjective norms, and perceived behavioral control for the TPB-SSB variables were somewhat better than TPB-PA variables. Since intervention implementation and program engagement were similar between conditions, this finding is most likely due to differences in the complexity, skills, and barriers involved in changing SSB versus PA behaviors. Given that the majority of TPB studies target a single behavior and are cross-sectional, there are few experimental studies to compare with our findings. As postulated, there was also a strong SSB media literacy effect. Media literacy, defined as an individuals’ ability to access, analyze, and evaluate media messages [45], was an underlying concept in the trial design for both conditions. The second small group class in both conditions combined motivational components and media skill-based training to prompt individuals to advocate for their own health [71]; however, the outcome measure focused exclusively on SSB media literacy. Although media literacy training has been used in adolescents, this is one of the first known adult trials to incorporate and evaluate media literacy skills [72]. Future analyses are needed to understand the causal pathways and interdependence of sociocognitive and psychological determinants (e.g., TPB constructs), skill-based factors (e.g., HL, media literacy), and demographic factors as they relate to behavioral targets.

With the exception of BMI, this trial did not result in between group effects for clinical outcomes. This finding is not entirely unexpected, since improvements in either behavior may improve these outcomes. Within SIP*smart*ER, improvements were noted for total cholesterol and low-density lipoprotein cholesterol, but not for blood pressure, glucose, or triglycerides. Within MoveMore, improvements were observed for low-density lipoprotein, but not for other clinical outcomes. Several explanations are plausible for these null findings on the secondary outcomes. First, the average baseline values generally indicate normal values, so there may have been less opportunity for meaningful and statistically significant improvements (i.e., abnormal clinical values were not an inclusion criteria). Second, the interventions may not have been of sufficient intensity, focus, or duration to produce changes in these more distal clinical outcomes. Nonetheless, having this experimental data available is of value, as the majority of literature related to excessive SSB consumption among adults is limited to observational studies [7, 63–68]. Future analysis should explore whether the magnitude of change in the behavioral targets (i.e., SSB and PA) influenced changes in clinical outcomes and examine differential changes among participants with high versus normal clinical values at baseline. Also, the 12 month maintenance phase of this trial is on-going through December 2015. Therefore, the maintenance of behaviors and long-term influence on clinical outcomes are not yet available, but forthcoming.

Health literacy did not influence retention, engagement, or outcomes. These findings fill an important gap in the HL body of research. Despite notable advancements in experimental approaches to address HL skills [11, 12], few experimental studies report on how participant HL status influences retention and engagement, or how HL moderates outcomes [14]. In one systematic review of experimental HL research articles, only 8 of 24 trials performed a moderation analysis by HL category and findings were mixed [14]. To fully advance HL research, concentrated efforts are needed to understand if research trials are exacerbating or reducing HL-related health disparity gaps. In an effort to achieve a balance between HL skills/ability and demands/complexity of the targeted behavior [73], a central goal in this line of research has been to apply health literacy strategies and to develop a culturally sensitive intervention for rural, underserved populations [16, 28, 29]. Importantly, our findings suggest that focused efforts and application of HL strategies resulted in similar engagement and benefits among low and high HL participants. Additional analyses are warranted to determine if changes in HL mediate changes in outcomes.

In addition to the aforementioned opportunities for further data analysis, and as guided by the RE-AIM planning and evaluation framework [15, 20], on-going efforts are underway to understand the context for adoption and implementation, including recruitment, intervention delivery costs, and post-program participant qualitative data to identify factors that influenced intervention engagement and success. Our preliminary data indicate that implementation fidelity was similarly high among both conditions. Participant-centered program evaluation data may provide insights into differences of behavior change and maintenance for SSB versus PA. Finally, given the relatively encouraging results of both the SIP*smart*ER and MoveMore interventions, as well as the emphasis and potential benefits of multiple behavior change interventions [74, 75], future efforts should explore combining programmatic elements to concurrently address both SSB and PA.

### Study Limitations

This study may have limited generalizability beyond the targeted medically-underserved Appalachian region of rural southwest Virginia. However, with the exception of men, this enrolled sample was generally representative of this high need region. Also, while this trial was powered to detect the relative 6 month treatment effect between the two conditions, it was not powered to detect HL moderation effects. Therefore, the non-statistically significant moderation effect findings should be interpreted with caution. Likewise, non-statistically significant secondary outcomes should also be interpreted somewhat cautiously, as lack of statistical power may be an issue. The self-reported measures may also be considered a study limitation; however, our unpublished 6 month C13 biomarker data, which entails technical details beyond the scope of this primary outcomes manuscript [76, 77], provides concurrent validity for meaningful improvements in SSB behaviors. Finally, while all analytic procedures for the treatment of missing data are subject to controversy, we are transparent in our analytical approach and present the most conservative 6 month results among the different analytic procedures with the goal to provide lower bound of program effectiveness assessment. To briefly illustrate this point, the Cohen’s d effect size for kcals/day of SSB was 0.55 for the provided intention-to-treat analysis (*p* < 0.001), and was 0.69 with the present at follow-up analysis (completers only) (*p* < 0.001). Despite these limitations, this trial allowed all participants an opportunity to benefit, while providing an adequate sample size to examine treatment effects on SSB consumption and allowing the opportunity to explore moderation effects and secondary outcomes.

## Conclusions

Our findings provide evidence that SIP*smart*ER is an effective behavioral and HL intervention to decrease SSB consumption among rural adults in a medically-underserved region. Grounded by a HL framework, our intervention purposefully focused on an important, yet relatively simple behavior—with a goal of achieving a balance between HL skills/ability and the demands/complexity of the targeted behavior [73]. Additional efforts are needed to understand long-term changes in anthropometric outcomes. Results also indicate adequate reach and engagement of the targeted population with concerted focus on scalable strategies that were also technologically, culturally, and literacy appropriate. Application of the RE-AIM planning and evaluation framework provided a key conceptual guide to help operationalize study outcomes, as well as direct methodological decisions that intended to simultaneously balance internal and external validity factors [15, 20]. To promote broader public health impacts of SSB reductions, additional efforts are needed to explore dissemination and implementation of SIP*smart*ER among local organizations that operate within rural and medically-underserved regions. Additionally, while environmental- and policy-level SSB approaches such as limiting consumer access and taxation appear promising [78–82], these strategies are often met with controversial opposition. As empirical data begins to emerge for strategies implemented and evaluated at higher levels (i.e., community, environmental, and policy) to reduce SSB intake and BMI status, further examination is need to understand the relative value and impacts of individual-level health behavior and HL strategies targeting SSB consumption, such as those used in SIP*smart*ER. The largest public health impacts will likely be realized when SSB reduction strategies are integrated across multiple levels of influence [82, 83].

## Abbreviations

BMI: Body mass index
HL: Health literacy
IVR: Interactive voice response
PA: Physical activity
SSB: Sugar-sweetened beverages
TPB: Theory of planned behavior

## References

[1] Kit BK, Fakhouri THI, Park S, Nielsen SJ, Ogden CL (2013). Trends in sugar-sweetened beverage consumption among youth and adults in the United States: 1999–2010. Am J Clin Nutr.

[2] Sharkey JR, Johnson CM, Dean WR. Less-healthy eating behaviors have a greater association with a high level of sugar-sweetened beverage consumption among rural adults than among urban adults. Food Nutr Res. 2011;55 http://www.ncbi.nlm.nih.gov/pmc/articles/PMC3153312/. 10.3402/fnr.v55i0.5819 PMC3153312 21845142

[3] Zoellner J, You W, Connell C, Smith-Ray RL, Allen K, Tucker KL (2011). Health literacy is associated with healthy eating index scores and sugar-sweetened beverage intake: findings from the rural lower mississippi delta. J Am Diet Assoc.

[4] Fung TT, Malik V, Rexrode KM, Manson JE, Willett WC, Hu FB (2009). Sweetened beverage consumption and risk of coronary heart disease in women. Am J Clin Nutr.

[5] Ismail AI, Sohn W, Lim S, Willem JM (2009). Predictors of dental caries progression in primary teeth. J Dent Res.

[6] Johnson RK, Appel LJ, Brands M, Howard BV, Lefevre M, Lustig RH (2009). Dietary sugars intake and cardiovascular health: a scientific statement from the american heart association. Circulation.

[7] Malik VS, Schulze MB, Hu FB (2006). Intake of sugar-sweetened beverages and weight gain: a systematic review. Am J Clin Nutr.

[8] Vartanian LR, Schwartz MB, Brownell KD (2007). Effects of soft drink consumption on nutrition and health: a systematic review and meta-analysis. Am J Public Health.

[9] Tate DF, Turner-McGrievy G, Lyons E, Stevens J, Erickson K, Polzien K (2012). Replacing caloric beverages with water or diet beverages for weight loss in adults: main results of the choose healthy options consciously everyday (CHOICE) randomized clinical trial. Am J Clin Nutr.

[10] Haynes MA, Smedley BD (1999). The unequal burden of cancer: an assessment of NIH research and programs for ethnic minorities and the medically underserved. institute of medicine.

[11] Berkman N, DeWalt D, Pignone M, Sheridan S, Lohr K, Lux L, et al. Literacy and Health Outcomes. Rockville, MD: Agency for Healthcare Research and Quality. RTI International-University of North Carolina Evidence-based Practice Center under Contract No. 290-02-0016; January 2004.

[12] Berkman ND, Sheridan SL, Donahue KE, Halpern DJ, Viera A, Crotty K (2011). Health literacy interventions and outcomes: an update of the literacy and health outcomes systematic review of the literature.

[13] U.S. Department of Health and Human Services. Healthy People 2010: Understanding and Improving Health. 2nd ed. Washington, DC: U.S. Government Printing Office, 2000.

[14] Allen K, Zoellner J, Motley M, Estabrook P (2011). Understanding the internal and external validity of health literacy interventions: a systematic literature review using the RE-AIM framework. J Health Commun.

[15] Curran GM, Bauer M, Mittman B, Pyne JM, Stetler C (2012). Effectiveness-implementation hybrid designs combining elements of clinical effectiveness and implementation research to enhance public health impact. Med Care.

[16] Zoellner J, Krzeski E, Harden S, Cook E, Allen K, Estabrooks PA (2012). Qualitative application of the theory of planned behavior to understand beverage consumption behaviors among adults. J Acad Nutr Diet.

[17] US Department of Commerce, US Census Bureau. State & County QuickFacts. http://www.census.gov/quickfacts/. Accessed 19 July 2013.

[18] Evenson KR, Herring AH, Huston SL (2005). Evaluating change in physical activity with the building of a multi-use trail. Am J Prev Med.

[19] Ajzen I (1991). The theory of planned behavior. Organ Behav Human Decis Process.

[20] Glasgow RE, Vogt TM, Boles SM (1999). Evaluating the public health impact of health promotion interventions: The RE-AIM framework. Am J Public Health.

[21] Virginia Medically Underserved Areas. http://www.vdh.state.va.us/OMHHE/primarycare/shortagedesignations/documents/Medically%20Underserved.pdf. Accessed 6 Dec 2015.

[22] What is Rural? 2014 https://ric.nal.usda.gov/what-rural. Accessed 1 September 1 2015.

[23] Hedrick VE, Savla J, Comber DL, Flack KD, Estabrooks PA, Nsiah-Kumi PA (2012). Development of a brief questionnaire to assess habitual beverage intake (bevq-15): sugar-sweetened beverages and total beverage energy intake. J Acad Nutr Diet.

[24] Thomas S, Reading J, Shephard RJ (1992). Revision of the physical activity readiness questionnaire (PAR-Q). Can J Sport Sci.

[25] Popkin BM, Armstrong LE, Bray GM, Caballero B, Frei B, Willett WC (2006). A new proposed guidance system for beverage consumption in the United States. Am J Clin Nutr.

[26] Glasgow RE (2013). What does it mean to be pragmatic? Pragmatic methods, measures, and models to facilitate research translation. Health Educ Behav.

[27] Centers for Disease Control and Prevention, Physical Activity for Everyone. http://www.cdc.gov/physicalactivity/basics/. Accessed 1 September 2014.

[28] Zoellner J, Estabrooks P, Davy B, Chen Y, You W (2012). Exploring the theory of planned behavior to explain sugar-sweetened beverage consumption. J Nutr Ed Behav.

[29] Zoellner J, Cook E, Chen Y, You W, Davy B, Estabrooks P (2013). Mixed methods evaluation of a randomized control pilot trial targeting sugar-sweetened beverage behaviors. Open J Prev Med.

[30] Estabrooks PA, Smith-Ray RL, Almeida FA, Hill J, Gonzales M, Schreiner P (2011). Move More: Translating an efficacious group dynamics physical activity intervention into effective clinical practice. Int J Sport Exerc Psychol.

[31] Estabrooks P, Downey S, Burke S (2012). Group dynamics in physical activity interventions: what works?. Soc Personal Psychol Compass.

[32] Estabrooks P (2000). Sustaining exercise participation through group cohesion. Exerc. Sport Sci. Rev.

[33] Porter K, Chen Y, Estabrooks P, Noel L, Baily A, Zoellner J. Using Teach-Back to Understand Participant Behavioral Self-Monitoring Skills Across Health Literacy Levels and Behavioral Condition. J Nutr Ed Behav. In Press. 10.1016/j.jneb.2015.08.012 PMC4715922 26453368

[34] Estabrooks PA, Smith-Ray RL (2008). Piloting a behavioral intervention delivered through interactive voice response telephone messages to promote weight loss in a pre-diabetic population. Patient Educ Couns.

[35] Estabrooks PA, Shoup JA, Gattshall M, Dandamudi P, Shetterly S, Xu S (2009). Automated telephone counseling for parents of overweight children- A randomized controlled rrial. Am J Prev Med.

[36] Estabrooks PA, Glasgow RE, Xu S, Dzewaltowski DA, Lee RE, Thomas D (2011). Building a multiple modality, theory-based physical activity intervention: the development of CardiACTION. Psychol Sport Exerc.

[37] Zoellner J, Chen Y, Davy B, You W, Hedrick V, Corsi T (2014). Talking Health, a pragmatic randomized-controlled health literacy trial targeting sugar-sweetened beverage consumption among adults: rationale, design and methods. Contemp. Clin. Trials.

[38] Armitage CJ, Conner M (2001). Efficacy of the theory of planned behaviour: a meta-analytic review. Br J Soc Psychol.

[39] Godin G, Kok G (1996). The theory of planned behavior: a review of its applications to health-related behaviors. Am J Health Prom.

[40] McEachan R, Conner M, Taylor N, Lawton R. Prospective prediction of healht-related behaviours with the Theory of Planned Behavior: a meta-analysis. Health Pyschology Review. 2011,5:1-48.

[41] Hardeman W, Johnston M, Johnston DW, Bonetti D, Wareham NJ, Kinmonth AL (2002). Application of the theory of planned behaviour in behaviour change interventions: a systematic review. Psychol Health.

[42] Golbeck AL, Ahlers-Schmidt CR, Paschal AM, Dismuke SE (2005). A definition and operational framework for health numeracy. Am J Prev Med.

[43] US Department of Health and Human Services, Health Resources and Services Administration. Effective Communication Tools for Healthcare Professionals. http://www.hrsa.gov/publichealth/healthliteracy/. Accessed 6 Dec 2015.

[44] Centers for Disease Control and Prevention, Health Literacy. http://www.cdc.gov/healthliteracy/developmaterials/. Accessed 6 Dec 2015.

[45] Aufderheide P (1993). Part II: conference proceedings and next steps. aspen institute report of the national leadership conference on media literacy.

[46] Bergsma LJ, Carney ME (2008). Effectiveness of health-promoting media literacy education: a systematic review. Health Educ Res.

[47] Estabrooks P, Glasgow R, Dzewaltowski D (2003). Physical activity promotion though primary care. JAMA.

[48] Weiss B, Mays M, Martz W, Merriam Castor K, DeWalt D, Pignone M (2005). Quick assessment of literacy in primary care: the newest vital sign. Ann Fam Med.

[49] Stote KS, Radecki SV, Moshfegh AJ, Ingwersen LA, Baer DJ (2011). The number of 24 h dietary recalls using the US Department of agriculture’s automated multiple-pass method required to estimate nutrient intake in overweight and obese adults. Public Health Nutr.

[50] Godin G, Shephard R (1985). A simple method to assess exercise behavior in the community. Can J Appl Sport Sci.

[51] Hedrick V, Comber D, Estabrook P, Savla J, Davy B (2010). The beverage intake questionnaire: determining initial validity and reliablity. J Am Diet Assoc.

[52] Riebl S, Paone A, Hedrick V, Zoellner J, Estabrooks P, Davy B (2013). The comparative validity of interactive multimedia questionnaires to paper- administered questionnaires for beverage intake and physical activity. JMIR Research Protocols.

[53] Kiernan M, Schoffman DE, Lee K, Brown SD, Fair JM, Perri MG (2013). The stanford leisure-time activity categorical item (L-Cat): a single categorical item sensitive to physical activity changes in overweight/obese women. Int J Obes.

[54] Rhodes RE, Courneya KS, Jones LW (2004). Personality and social cognitive influences on exercise behavior: adding the activity trait to the theory of planned behavior. Psychol Sport Exerc.

[55] Primack BA, Gold MA, Switzer GE, Hobbs R, Land SR, Fine MJ (2006). Development and validation of a smoking media literacy scale for adolescents. Arch Pediatr Adolesc Med.

[56] Centers for Disease Control and Prevention. Health-Related Quality of Life. http://www.cdc.gov/hrqol/concept.htm. Accessed 19 July 2013.

[57] Perloff D, Grim C, Flack J, Frohlich ED, Hill M, McDonald M (1993). Human blood-pressure determination by sphygmanometry. Circulation.

[58] Clinical Performance of the CardioChek P.A. and the Cholestech LDX System Compared to a Clinical Diagnostic Laboratory Reference Method for the Determination of Lipid Profiles. Technical Brief. http://www.hmscweb.com/PDF_Files/Cholestech/Technical_Brief_LDX_-_Polymer_CardioChek-PA_-_MKT_12508_Rev.A.pdf. Accessed March 2016.

[59] Rubin DB (1987). Multiple imputations for nonresponse in surveys.

[60] Laird N, Ware J (1982). Random effects models for longitudinal data. Biometrics.

[61] Raab GM, Day S, Sales J (2000). How to select covariates to include in the analysis of a clinical trial. Contemp. Clin. Trials.

[62] Borenstein M, Cooper H, Hedges LV, Valentine JC (2009). Effect sizes for continuous data. The handbook of research synthesis and meta-analysis.

[63] Hu FB (2013). Resolved: there is sufficient scientific evidence that decreasing sugar-sweetened beverage consumption will reduce the prevalence of obesity and obesity-related diseases. Obes Rev.

[64] Kaiser KA, Shikany JM, Keating KD, Allison DB (2013). Will reducing sugar-sweetened beverage consumption reduce obesity? Evidence supporting conjecture is strong, but evidence when testing effect is weak. Obes Rev.

[65] Olsen NJ, Heitmann BL (2009). Intake of calorically sweetened beverages and obesity. Obes Rev.

[66] Malik VS, Willett WC, Hu FB (2009). Nutritively sweetened beverages and obesity. JAMA.

[67] Pan A, Malik VS, Hao T, Willett WC, Mozaffarian D, Hu FB (2013). Changes in water and beverage intake and long-term weight changes: results from three prospective cohort studies. Intern J Obes.

[68] Malik VS, Pan A, Willett WC, Hu FB (2013). Sugar-sweetened beverages and weight gain in children and adults: a systematic review and meta-analysis. Am J Clinic Nutr.

[69] Conn VS, Hafdahl AR, Mehr DR (2011). Interventions to increase physical activity among healthy adults: meta-analysis of outcomes. Am J Public Health.

[70] Brownson R, Colditz GA, Proctor E (2012). Dissemination and implementation research in health: translating science to practice.

[71] Austin EW, Pinkleton BE, Van de Vord R, Arganbright M, Chen Y-CY (2006). How orientations toward media use affect media literacy outcomes in a test focused on channel one news. Acad Exch J.

[72] Hindin TJ, Contento IR, Gussow JD (2004). A media literacy nutrition education curriculum for head start parents about the effects of television advertising on their children’s food requets. J Am Diet Assoc.

[73] IOM (Institute of Medicine). Measures of Health Literacy. Workshop Summary. Washington, DC: The National Academies Press; 2009. 20845551

[74] Prochaska J, Prochaska J (2011). A review of multiple health behavior change interventions for primary prevention. Am J Lifestyle Med.

[75] Prochaska JJ, Nigg CR, Spring B, Velicer WF, Prochaska JO (2010). The benefits and challenges of multiple health behavior change in research and in practice. Prev Med.

[76] Hedrick V, Zoellner J, Woodford N, Jahren H, Bostic H, Davy B (2015). A dual-carbon-and-nitrogen stable isotope ratio model is not superior to a single-carbon stable isotope ratio model for predicting added sugar intake in southwest virginian adults. J Nutr.

[77] Hedrick V, Davy B, Wilburn Jahren H, Zoellner J (2015). Evaluation of a novel biomarker of added sugar intake (δ 13C) compared with self-reported added sugar intake and the Healthy Eating Index-2010 in a community-based, rural US sample. Public Health Nutr.

[78] Greathouse KL, Chriqui J, Moser RP, Agurs-Collins T, Perna FM (2013). The association of soda sales tax and school nutrition laws: a concordance of policies. Public Health Nutr.

[79] Novak N, Brownell K (2011). Taxation as prevention and as a treatment for obesity: the case of sugar-sweetened beverages. Curr Pharm Design.

[80] Chriqui JF, Chaloupka FJ, Powell LM, Eidson SS (2013). A typology of beverage taxation: multiple approaches for obesity prevention and obesity prevention-related revenue generation. J Public Health Policy.

[81] Edwards RD (2011). Commentary: soda taxes, obesity, and the shifty behavior of consumers. Prev Med.

[82] McLeroy KR, Bibeau D, Steckler A, Glanz K (1988). An ecological perspective on health promotion programs. Health Educ Q.

[83] Egger G, Swinburn B (1997). An “ecological” approach to the obesity pandemic. BMJ.

